# Editorial: Promoting health management in rehabilitation

**DOI:** 10.3389/fresc.2025.1626049

**Published:** 2025-06-11

**Authors:** Jaclyn Schwartz, Elena Donoso Brown, Scott Bleakley

**Affiliations:** ^1^ Program in Occupational Therapy, Washington University in St. Louis, St. Louis, MO, United States; ^2^ Department of Occupational Therapy, Duquesne University, Pittsburgh, PA, United States; ^3^ UPMC Rehabilitation Institute, Pittsburgh, PA, United States

**Keywords:** intervention development, health management, disability, chronic disease, feasibility, rehabilitation

**Editorial on the Research Topic**
Promoting health management in rehabilitation

The prevalence of disability continues to increase globally, with approximately 25% of adults in the United States living with a disability (1). While medical advances have extended lifespans for people with disabilities, significant health disparities persist (2). People with disabilities experience substantially worse health outcomes compared to their non-disabled peers, including higher rates of cardiac disease, diabetes, and asthma (3). These disparities have led to the recent designation of people with disabilities as a health disparity population by both the National Institutes of Health (4) and Healthy People 2030 (5).

As rehabilitation practitioners, we are charged with the mandate to help people with disabilities achieve health equity. While rehabilitation has historically focused on functional recovery and independence in activities of daily living, our role must evolve to include supporting health management across the lifespan. While health interventions for people with disabilities are increasing, there are insufficient interventions to meet demand, and the interventions that do exist fail to leverage implementation science approaches to best address the most common barriers experienced by people with disabilities in their journey for better health (6).

In response to this growing need, we present this Research Topic, “*Promoting health management in rehabilitation*,” which brings together contributions that advance our understanding of how to develop and implement interventions that promote health and wellness in people with disabilities. The articles in this Research Topic align with various stages of the National Institute of Health Stage Model for Behavioral Intervention Development, offering insights into the complete intervention development process (7).

First, Starosta et al. explored the importance of peer-led self-management in creating community connections for people with spinal cord injury (SCI). Their qualitative analysis of online forum posts revealed that participants engaged in skill building, resource sharing, and problem solving. Most notably, they identified a critical process theme called “bearing witness,” describing the emotional connection to others with similar SCI-related challenges. Many participants reported that these groups represented their first engagement with a community having lived experience with SCI, highlighting the power of shared experience in making intervention content more impactful and relevant.

Gahlot et al. conducted a qualitative study exploring clinician and patient experiences with shared decision-making (SDM) to promote daily arm use for individuals with chronic stroke. They identified three key themes: equal partnership, enhancing clinician confidence, and the distinctiveness of SDM compared to traditional approaches. The study highlighted important facilitators, such as clinician competence with SDM and open, trusting relationships with patients, while also identifying barriers, including limited clinician expertise in SDM and patients' lack of foundational knowledge about stroke rehabilitation.

Addressing persistent pain management, Curran et al. described a collaborative care (CC) intervention called TBI Care, specifically adapted to treat chronic pain in people living with traumatic brain injury (TBI). This approach emphasized expert clinician input, cognitive behavioral therapy techniques, and other non-pharmacological strategies for decreasing pain interference. Their findings showed high participant engagement, with 90% of participants receiving at least 11 of the targeted 12 sessions. All participants received pain education, self-monitoring skills, goal setting/behavioral activation, and relaxation training, with significant decreases in pain interference scores throughout the intervention.

Schmid et al. conducted a pilot study to establish the feasibility and acceptability of a yoga and self-management education intervention called MY-Skills designed to support caregiving dyads (caregivers and care receivers) experiencing persistent pain. The intervention combined yoga with self-management education specifically developed for caregiving dyads. Their findings demonstrated that both in-person and online versions of the MY-Skills intervention exceeded benchmark criteria for attendance, safety, acceptability, and completion, with participants reporting high satisfaction despite the majority having experienced pain for over 10 years.

Finally, Donoso Brown et al. conducted a qualitative study exploring caregiver perceptions of usual care home programs for persons with acquired brain injury after transition to the community. They identified two key themes: (1) how systems, roles, and responsibilities influenced caregivers' perceptions of home programs and recovery outlook and (2) caregivers’ home program experience. Their findings revealed that caregivers' outlook on recovery and home program implementation was influenced by the burden of responsibilities and system-level supports and barriers. Though caregivers reported limited training, they found it satisfactory, saw value in home programs, and advised others to engage in them.

Collectively, these contributions advance our understanding of how to effectively develop and implement health management interventions for people with disabilities that match the needs identified by the community. The interventional approaches presented in this special issue demonstrate a shift toward more comprehensive, person-centered rehabilitation that extends beyond traditional time-limited interventions. This shift acknowledges that the journey of disability is often lifelong, requiring ongoing support for health management. By focusing on developing sustainable health behaviors, we can help individuals with disabilities not only manage their conditions but also potentially reduce the impact of secondary health complications.

Despite these promising advances, significant challenges remain. Implementation of health management interventions in real-world settings requires addressing systemic barriers, including limited insurance coverage for preventive services, healthcare provider knowledge gaps, and accessibility issues (2). Future research must continue to bridge the gap between intervention development and implementation science to ensure these innovative approaches reach those who need them most.

The path toward health equity for people with disabilities requires concerted efforts to develop, implement, and evaluate interventions that empower individuals to establish and maintain health-promoting behaviors. This Research Topic represents an important step in this journey, providing valuable guidance for researchers and clinicians alike. We hope these contributions will inspire continued innovation in this critical area of rehabilitation science.
